# Comparing Trawl and Creel Fishing for Norway Lobster (*Nephrops norvegicus*): Biological and Economic Considerations

**DOI:** 10.1371/journal.pone.0039567

**Published:** 2012-07-25

**Authors:** Ana Maria Leocádio, David Whitmarsh, Margarida Castro

**Affiliations:** 1 Faculdade de Ciências e Tecnologia, Universidade do Algarve, Campus Gambelas, Faro, Portugal; 2 Department of Economics, Portsmouth Business School, University of Portsmouth, Portsmouth, United Kingdom; 3 CCMAR, Centre of Marine Sciences, Universidade do Algarve, Campus Gambelas, Faro, Portugal; Swansea University, United Kingdom

## Abstract

This study compares the fishing activity and landings of the trawl and creel fisheries for Norway lobster (*Nephrops norvegicus* (L.)) off the Portuguese coast, and evaluates the financial viability of two vessels typical of each fleet. Crustacean trawlers are part of an industrial fleet that, besides *Nephrops*, targets deep water shrimps. Creels are used by a multi-gear, multi-target artisanal fleet, fishing only in areas unavailable to trawlers and, when catching *Nephrops*, set specifically to target this species. Trawlers have in recent years contributed with 85% of the landings in weight, but only 74% in value (2005–2009 average). Despite smaller landings, the *Nephrops* creel fishery provides individuals of larger size and in better condition, thereby obtaining higher unit prices. Economic viability was also higher for the creel vessel, with trawling being only viable if major costs (such as labor and fuel) are covered by the revenue from other target species (e.g., the rose shrimp). At present, *Nephrops* populations on the South and SW coast are subject to intense fishing and to a recovery plan. The possibility of reallocation of some of the fishing effort directed at *Nephrops* from trawlers to creels is discussed in terms of the conservation of the resource and economic return.

## Introduction

The Norway lobster (Nephrops norvegicus (L.)) is the most valuable invertebrate resource caught in EU waters with a total first sale annual value of around 431 million Euros, corresponding to 74 thousand tonnes for the 2007–2008 average (EUROSTAT, http://epp.eurostat.ec.europa.eu/portal/page/portal/fisheries/data/database). In 2008 the UK and Ireland accounted for over 70% of the landings; Portugal ranked 13th with estimated landings of 247 tones (EUROSTAT). Throughout Europe, this species is caught mainly by trawling. Despite the relatively minor importance of Portuguese landings in the context of global European catches, first sale values are considerably higher in Portugal than elsewhere in Europe. While the average price per kg in 2008 was 7.51 € for Europe, it was 23.95 € in Portugal. This is in part due to the quality of the product; Norway lobsters are sold either fresh, refrigerated with ice (from the trawling fleet), or alive (from the artisanal fleet).

**Figure 1:**
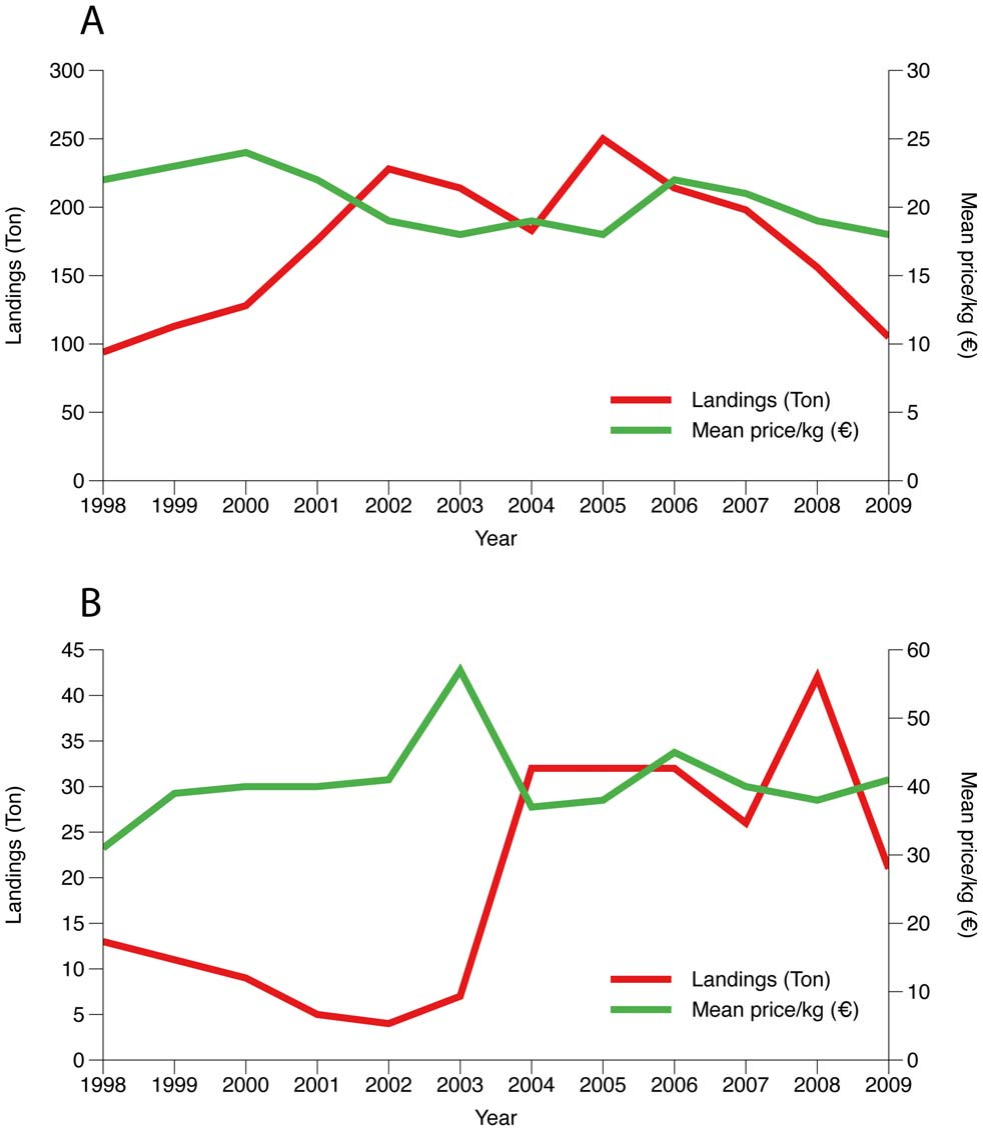
Evolution of the landings in weight and value. Annual landings in tons and mean price per Kg, for the trawl (A) and creel (B) fleets landing *Nephrops* , 1998–2007. Trawl landings exceed creel landings in weight. However, creel landings always obtained higher prices per Kg. Prices were converted from nominal to real terms using the Harmonized Index of Consumer Prices (HICP at EUROSTAT [15]).

Portuguese Norway lobster landings have decreased abruptly from annual totals of around 1500 tons in 1987 to 329 tons in 1989. Subsequently, reported landings have not recovered to previous levels, staying below 400 tons since the 1990s (Figure 1A) (DGPA - General Directorate for Fisheries and Aquaculture, statistical information, http://www.dgpa.min-agricultura.pt/portal/page?_pageid=33,46256&_dad=portal&_schema=PORTAL&g_d=11050&cboui=11050). Management recommendations are produced by ICES (International Council for the Exploration of the Sea) and the assessment carried out in 2001 [1] considered that this drop in landings, since the 1980s, was the result of a severely declining stock, due to over-exploitation. A recovery plan was approved in December 2005 (Council Regulation (CE) N° 2166/2005) imposing limits on the caches and effort and defining closed areas. A minimum 70 mm diagonal mesh is enforced for codends of trawlers targeting Norway lobster and there are no requirements for selective devices in the trawling nets. A month closure (January) is imposed the operation of this fleet is also restricted under a general regulation that prohibits trawling in areas within 6 miles from the shore. The minimum landing size for Nephrops is 20 mm carapace length. The limitations to creeling are the number of traps per vessel (varying from 500 to 1000 according to the length of the vessel), the mesh size, that when targeting Nephrops is 30 to 50 mm square mesh or a 40 mm distance between bars and forbiddance of landing berried females. The most common traps used for Nephrops are built with an iron frame wrapped with 40 mm rigid plastic netting.

In Portugal most of Nephrops landings came from the crustacean trawling fleet, composed at present of approximately 40 vessels that along the last two decades have transferred part of the fishing effort towards the rose shrimp (Parapenaeus longirostris), today the most important species, and the red shrimp (Aristeus antenatus). This change in effort accounts, in part, for the reduction in Nephrops landings, but unfortunately no specific studies exist that allow the quantification of effort changes.

**Figure 2:**
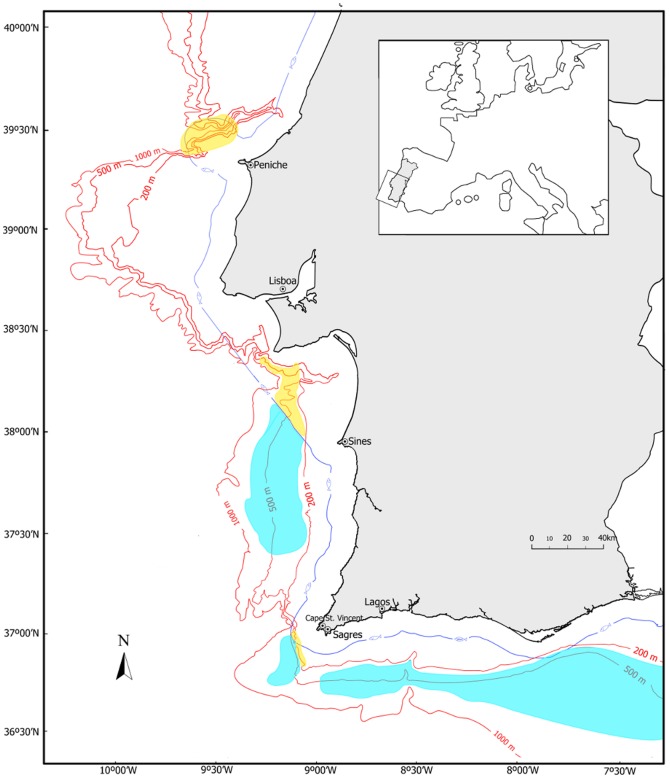
Map showing the location of the fishing grounds. Traditional fishing grounds are shown: fishing grounds explored with trawling in blue and creeling in yellow. Sampling for this work was done NE of Peniche (creels) and off Lagos on the south coast (trawl). Sources of information for mapping the fishing grounds: [32]–34.

Creels were introduced roughly 30 years ago and have since accounted for a small proportion of total landings, but from 2003 onwards (Figure 1B), the importance of this fishery increased, reaching and maintaining levels of around 15% of the landings in weight and over 26% in value (average 2004–2009 DGPA database). At present, there are roughly 10 artisanal vessels that use creels (DGPA database), baited usually with mackerel or bogue (100–150 g per creel and ∼0.30€ per kg). Creeling in restricted to areas where trawlers cannot operate. These are areas with large boulders on muddy bottoms and areas within 6 miles from the coast. In these areas, very large Nephrops support a highly valuable creel fishery that markets live individuals. All the creeling fishing grounds are located on the southwest coast (Figure 2). In the South coast, the lack of trawl free sites has discouraged the practice of creeling. In Europe, creels have been implemented locally to fish Nephrops in some inshore areas around the west coast of Scotland [2], Sweden [3], north-eastern Adriatic and Ionian Sea [4] and since 1980, after the complete prohibition of trawling, in inshore areas, off the Faeroe Islands [5].

In face of the possibility that present levels of Nephrops trawling may be excessive, resulting in recommendations to stop or decrease catches [6]–[14], the use of creels as an alternative should be seriously considered. This is the rationale for the present work, which makes a comparative assessment of the biological and economic characteristics of both gear types, by thoroughly investigating the fishing pattern, composition of the catches, values of costs and returns for one trawler and a vessel using creels, representing well their fleets.

## Materials and Methods

### Ethics statement

No specific permits were required for the described field studies. Data resulted from the observation of fisheries catches, onboard fishing vessels, at port landings or at fish auctions. In all cases the sampled individuals were obtained through regular fishing operations, our sampling only involved the measurements of carapace length and sex identification.

The characteristics of the trawl and creel *Nephrops* fisheries considered were: fishing effort targeting *Nephrops*, catch information (structure, condition and value) and financial information. In addition to global values for both fleets, a typical vessel from each one (with average catches similar to those of the fleet they represent) was chosen, and as much information as possible was gathered for each one. For these two vessels, detailed information regarding biological and economic aspects was obtained, including on board and at the port sampling of the landings, and extensive interviews with crew members and managers of the fishing companies that own the vessels.

### Fishing activity and landings

General characteristics of the trawl and creel *Nephrops* fishing are presented in Table 1. Information on the Portuguese commercial fisheries was obtained from the DGPA database, and includes individual data by vessel: type of fishing license, total number of trips per year, landings per trip, gross revenue per trip, species landed per trip and fishing port used for landing. This information is given on a trip-by-trip basis, between 1998 and 2007. The sale values of landings (Euros), presented in nominal terms, were converted to real terms using the Harmonized Index of Consumer Prices (HICP at EUROSTAT [15]). The HICPs, are economic indicators constructed to measure the changes over time in the prices of consumer goods and services acquired by households, and provide the official measure of consumer price inflation in the euro-zone [15].

**Table 1:** Main differences between trawl and creel *Nephrops* fishery.

	Trawl fishery	Creel fishery
Vessels size (meters)	>25	<20
Fuel consumption	High	Low
Number of vessels	∼30	∼10
Area	Offshore (6 miles limit)	Areas inaccessible to trawling
Duration of fishing trip (days)	1 to 3	1 (usually once a week)
Target species	Mixed crustacean fishery	Selective for *Nephrops*
Selectivity	Low	High
Discards (of total catch)	>60%(1)	Almost non-existent(2)
Environmental impacts	High(3)	Low(4)

Source: DGPA database,

(1) [29]–[31],

(2) [4], [28], [35], [36],

(3) [37]–[41] and,

(4) [25], [27], [28].

On board sampling of the trawler's catches occurred during summer 2007, on the south coast of Portugal at depths ranging from 350 to 650 m, using commercial nets with 70 mm cod-end mesh size. Catch structure for the vessel using creels was obtained at several landing ports on the southwest coast, also during the summer of 2007. Catches were obtained at depths ranging from 600 to 650 m using 1000 creels with 40 mm mesh size. Biological sampling included the measurement of standard length with calipers, (carapace length measured in mm). During biological sampling, the condition of *Nephrops* caught was measured by a scale indicating vitality [16], defined from 0 to 2 (0 = no signs of life; 1 = some movement of the appendages; 2 = strong signs of life, assuming aggressive posture).

*Nephrops* is sold at the fish auction of Vila Real de Santo António port, sorted into 4 size categories of different value, category 1 being the largest and most expensive and 4 the smallest and cheapest. Landings already sorted by size category were sampled at the fish auction in June and July of 2007. The probability of a length class falling into a given size category was estimated and extrapolated to the catches sampled in this work.

### Financial viability

The basic criterion used to assess the financial sustainability of the trawl and creel fishery was based on a measure of financial viability of capital investment, the net present value (NPV). NPV is defined as the present value of net cash flows. It is a standard method for using the time value of money to appraise long-term projects [17].

For both trawls and creels only a part of the fishing effort is allocated to *Nephrops*. For the trawler it was estimated that 1/3 of the fishing effort was allocated to *Nephrops* (based on proportion of trawling time targeting *Nephrops*). For the creel vessel the effort allocated for *Nephrops* is estimated to be 1/5 of the total fishing effort (proportion of days fishing this species).

The financial viability of the two vessels, for the *Nephrops* fishery only, was compared using the net present value (NPV), representing the financial worth of investment of fishing (comparisons of costs and revenues). The NPV was calculated using the expression:
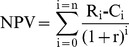
Where R_i_ is the revenue earned in year i, C_i_ is the costs incurred in year i, r is the discount rate (representing the opportunity of capital) and n is the investment time horizon in years.

The time horizon considered was of 10 years. The 5% discount rate used was based on recommendations from the European Commission [18] to determine NPV. The input data used to calculate the NPV was obtained for the sampled vessels for the year 2007, by interviewing the owners and captains and complemented with information extracted from the DGPA landings database. Data includes running costs (proportional to vessel utilization rate), fixed costs (those incurred regardless of the amount of fishing activity) and labor costs based on a share of net revenue (gross revenue minus running costs) for the artisanal fishery, and a fixed value for the trawl fishery. Data regarding price per kg, fishing days per year and catch rate (kg per fishing day) were also included.

The decision regarding which costs and revenues should be included was entirely based on the demands of *Nephrops* fishery: fuel (fishing and travelling) and lubricants for trawlers and bait and fuel (traveling) for the creel fishing. Other costs were allocated to *Nephrops* fishing proportionally to the fraction of the fishing effort dedicated to catch *Nephrops* (1/3 for the trawler and 1/5 for the creel vessel).

Sensitivity analysis was conducted to assess the financial viability of the *Nephrops* fishery in both vessels by changing the variables that affect NPV one at a time [17]. The results of the sensitivity analysis are summarized in a switching value, the level of change in a given variable required for the NPV to become zero, under the assumption that all other variables remain at present values.

## Results

### Characterization of the landings

The technical characteristics of the two vessels under study were obtained from the DGPA and are presented in Table 2. The landings of individual vessels were analyzed in detail for the years 2005–2007 in order to understand how representative of their fleets were the two vessels chosen.

**Table 2:** Technical characteristics of the vessels selected.

Characteristics	Trawl vessel	Creel vessel
Crew (number)	6–7	6
Total length (meters)	24.80	17.08
Engine (HP)	600	134.23
Gross tonnage	215	26.71
Construction year	2000	1993

Source: DGPA database.

Of the 35 trawlers that landed Nephrops, the fleet targeting this species was considered to be composed of vessels that simultaneously satisfied two criteria: the proportion of crustaceans in the landings was over 15% and the total amount of Nephrops was over one ton per year. A total of 17 vessels satisfied both criteria (accounting for 73% of Nephrops trawling landings), with average annual landings from 3.4 to 16.7 tons in weight and 54 and 323 thousand Euros in value. For the same period, the trawler selected for this work had average annual landings of 8.4 tons with a value of 202 thousand Euros. The artisanal fleet that landed Nephrops between 2005 and 2007 was composed of 29 vessels. It was considered that the fleet targeting Nephrops included all the vessels with landings in the three years, a total of 5 units. Two more were added that, despite landing Nephrops for only two of the years considered, had average landings of over one ton per year and represented 58% of the landings in those years. For the combination of 2005 to 2007, these 7 units were responsible for over 90% (both in weight and value) of the Nephrops catches landed by the artisanal fleet. The range of annual landings for these vessels was 1.0 to 13.2 tons and 31 to 367 thousand. The artisanal vessel selected had average landings of 2.9 tons in weight and 248 thousand Euros in value. Overall landings (for all vessels engaged in the Nephrops fishing) and mean annual prices per kg, for the last 12 years with available data, are given in Figure 2. Trawling landings, after an increase until 2002, remained above average until 2005 and have since decreased to the low values observed at the beginning of the period considered here. In 2009 the Nephrops landings of the crustacean fleet were around 100 tons. For the creel fishery, landings rose sharply until 2003 and have remained high until 2006, and suffered a drop from 42 to half that value afterwards. This reduction did not affect individual vessels yields and was due to the removal of 2 of the most important vessels from this fleet segment (DGPA database).

**Figure 3:**
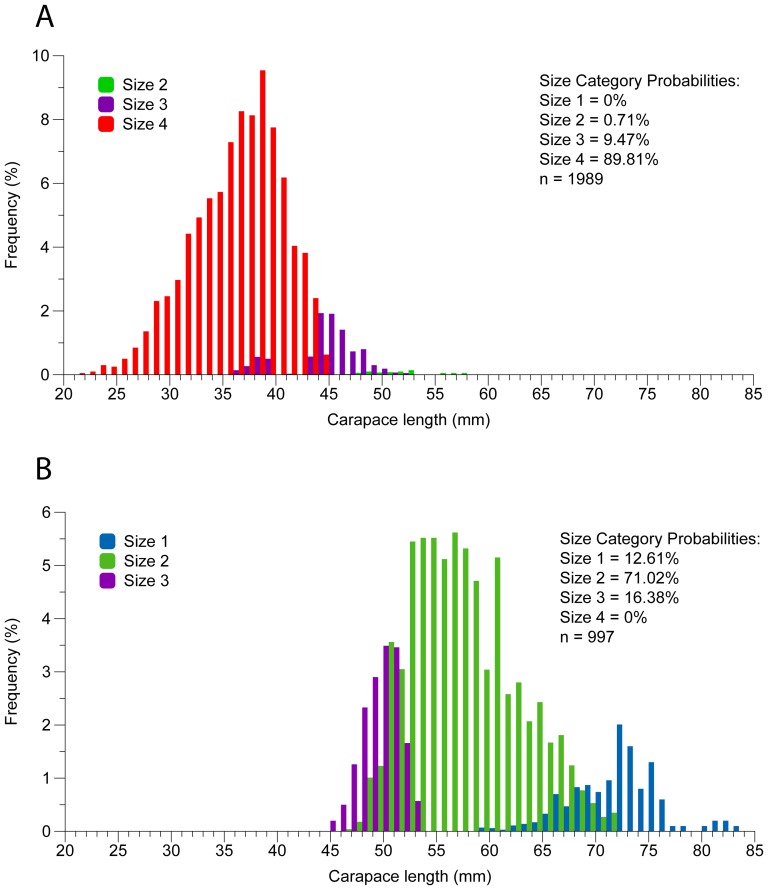
Size structure of the landings. Size frequency distribution (in percentage) of trawl (A) and creel (B) catches, including size probabilities for each size category. Trawl catches are composed of small individuals while creel catches are composed of large individuals. Ninety percent of the trawl catches are traded in size category 4 (lowest value) and category 1 (highest value) is absent. On the other hand, 13% of the creel catches are sold in size category 1, while 71% are in size category 2 and 16% in size category 3. The size distributions for each size category were sampled at the Vila Real de Santo António fish auction and result from the onboard separation of the catch.

The length distributions and size categories of the catches for the sampled vessels are presented in Figure 3 and values for first sale in Table 3. The structure of the catches combines information from the size distributions obtained on board and from the length categories, obtained at the VRSA fish auction. Trawl catches were composed of small individuals (ranging from 20 to 58 mm carapace length, with a mean size of 37 mm) while creel catches were composed of large individuals (ranging from 45 to 84 mm with a mean size of about 58 mm). Ninety percent of the trawl catches were traded in size category 4 (lowest value, Table 3), and category 1 (highest value) was absent while 13% of the creel catches were sold in category 1, 71% were in category 2 and 16% in category 3. Regarding the conditions upon arrival on deck (Table 4) in trawl catches 16% of the individuals had no sign of life (39% with some movements and 45% very active) while creel caught individuals all arrived on deck showing clear signs of life (30% with some movements and 70% very active).

**Table 3:** Approximate price and corresponding size ranges for *Nephrops*.

Size categories	CL (mm)	Approximate range (€/Kg)
1	>60	47–130(*)
2	47–72	23–67
3	36–53	7–24
4	<44	2–10

Shows range of values of first sale (Euros/kg/size category) and carapace length (mm).

Source: data collected at the auction market of Vila Real de Santo António (VRSA) in June and July of 2007.

(*) can reach up to 200€/kg at particular times.

**Table 4:** Condition of *Nephrops* at arrival on deck.

Condition scale	0	1	2
Trawl	16 (2.7)	39 (3.6)	45 (3.6)
Creels	0	30 (6.3)	70 (6.3)

Description of the condition (in percentage) on arrival on deck for trawl (net mesh size = 70 mm, n = 186) and creel catches (net mesh size = 40 mm, n = 53). Condition scale: 0 = no movements, 1 = some movements, 2 = strong life signals (aggressive posture). Standard error values are in parenthesis.

### Economic valuation

The annual average selling price of Norway lobster (per kg) landed in 2007 was very different for the studied vessels: 15€ for the trawler (standard deviation 5.8) and 80€ for the creel vessel (standard deviation 20.8). The catch per unit effort (kg/day of fishing) was 26 kg for the trawler and 70 kg for the creel vessel. The trawler landed Nephrops 198 days against 45 days for the artisanal vessel. The annual running cost was 76 545€ for the trawler (sum of fuel and lubricants) and 18 000€ for the artisanal vessel (sum of fuel and bait), with total annual fixed costs of 35 457€ and 12 000€ respectively (Table 5). In 2007, the average fuel consumption was 2134 liters per day for the trawler and just 238 liters per day for the creel vessel.

**Table 5:** Baseline values used in the Net Present Value (NPV) analysis and switching values from sensitivity analysis.

Variable	Units	Present	Switching value
Creels			
Price	€ per kg	80.19	53.53
Vessel utilization	Fishing days per year	45	30
Catch rate	kg per day fishing	70.5	47.1
Running costs	€ per day	400	2 279
Fixed costs	€ per year	12 000	96 569
Labor costs	N/A		
Crew share	proportion net revenue	0.50	0.94
Financial support (subsidies)	N/A		
Taxes and Social security	Proportion gross revenue	0.17	0.83
NPV (with discount rate at 5%)	€	737 585	
Trawl			
Price	€ per kg	14.84	32.56
Vessel utilization	Fishing days per year	198	434
Catch rate	kg per day fishing	26.1	57.4
Running costs	€ per day	387	−57
Fixed costs	€ per year	35 457	−56 255
Labor costs	€ per year	69 714	−21 998
Crew share	N/A		
Financial support (subsidies)	€ per year	13 217	104 930
Taxes and Social security	N/A		
NPV (with discount rate at 5%)	€	−799 893	

NPV is the present value of net cash flows and the switching values summarize the sensitivity analysis, representing the value required for the NPV to become zero, under the assumption that all other variables remain unchanged.

Source: landings data from DGPA database, economic information provided by the companies owning the vessels selected.

The total benefit of fishing for Norway lobsters with creels was estimated at 737 585 € considering the NPV over 10 years at 5% discount rate. With the same criterion, Nephrops trawling was an unprofitable fishery, with losses of the order of 799 893 €. However, when the NPV for the trawler was estimated for the total catch, where the rose shrimp represents the most important fraction, there were considerable financial gains, with a benefit estimated at 1 041 201 €. The sensitivity of these results to the input variables was tested based on the switching value (Table 5). Results reveal that profitability for creels was not marginal and that this fishery would still be profitable with a drop by 30% of any one of the revenue-determining variables (price, catch rate, or vessel utilization). For the trawler, by contrast, any one of these variables would need at least to double in order for the Nephrops component of its activity to generate a positive NPV.

Table 6 shows values indicating the importance of the Nephrops in weight (10.4% for the creels and 8.5% for the trawl) and value (51.6% for the creels and 12.7% of the trawl) and the profitability of the activity using as indicators net revenue per kg Nephrops caught. Fishing for Nephrops with the trawler was not a profitable operation, with losses of 20.29 euros per kg of Nephrops caught. These losses decreases to 6.82 if labor costs not considered (totally allocated to the shrimp part of the operation).

**Table 6:** Importance of *Nephrops* in the fishery and efficiency of the gears.

Indicators	Creels	Trawl
Proportion *Nephrops* in weight	10.4%	8.5%
Proportion *Nephrops* in value	51.6%	12.7%
Net revenue/Kg *Nephrops* landed (Euros)	26.66	−17.72
Net revenue/Kg *Nephrops* landed (no labor) (Euros)		−4.25

Importance of *Nephrops* in weight and value and revenue per kg of *Nephrops* caught for 2007. For the trawler net revenue was calculated with and without labor costs.

## Discussion

When comparing the two gears used to catch *Nephrops* off the Portuguese coast, through a detailed analysis of two vessels representing trawling and creel fishing, marked differences can be observed in exploitation pattern and size and condition of the landings. In both cases *Nephrops* is only targeted for part of the time (roughly 1/5 of the effort for the creel vessel and 1/3 for the trawler). For the trawler, *Nephrops* fishing was subsidiary with respect to the main target species, the rose shrimp while for the artisanal vessel, fishing *Nephrops* with creels was an essential valuable complement.

The unit value of landings for *Nephrops* caught by creels is superior to that for trawls, due to the larger size and better condition of the individuals (sold alive), as was verified in other studies comparing the same gears [19], [20]. Trawls tend to capture a wide range of sizes, including small individuals down to the minimum landing size (20 mm CL), while no large animals (above 60 mm CL) were caught, probably due to the exhaustive exploitation of the south coast stock. Creels captured few animals below 45 mm CL.

The differences in size structure may result from two sources: level of exploitation of the stocks and behaviour when interacting with the gear. Higher fishing mortality and smaller size of first capture in trawling are likely to result in the disappearance of large sizes. Trawling catches are influenced by biological rhythms of burrow occupation and emergence, since *Nephrops* are caught when out of their burrows [21]–[23]. Creel catches are not influenced by burrow emergence patterns in the same way as trawl catches, but are still dependent on individual animal behavior likely related to feeding activity when attracted to bait in creels [24].

Observations with video [25] showed that larger individuals were more likely to enter further into the creel eye and be trapped. Similar results were observed in an aquarium study were the largest *Nephrops* consistently displaced smaller individuals and were the only ones to enter the creel [26]. It is also possible that dominance and aggressive behavior of larger animals inhibits smaller individuals from entering the creels or force them to escape through the mesh (rigid plastic square mesh with 4 cm side).

The presence of large sizes in creel catches may indicate that these populations are subjected to lower mortality rates than those from areas were trawlers operate.

The impact of creeling on benthic communities is minimal compared to trawling [25], [27], [28], an aspect relevant for conservation of biodiversity. Creels are very selective for the target species, catching almost exclusively *Nephrops* [25] while trawling produces large quantities of by-catch composed mainly of fish [29]–[31]. The discarding of targeted crustacean species is usually due to damage of the individuals in the net and/or during the sorting process. Nine percent of *Nephrops* are discards in the trawl fishery [31].

The financial analysis revealed a non-profitable trawl *Nephrops* fishery. On the other hand, the fishing the Norway lobster with creels was economically viable. Results from this study suggest that if the trawling fleet depended more on fishing *Nephrops*, the profitability of fishing activity would be compromised. On the other hand, a fishing day targeting *Nephrops* by an artisanal vessel should be very profitable. Here, costs are low and revenues are high; for the sampled vessels the fuel consumption is low (9 times less when compared with the trawler) and mainly justified by the journey to and from the fishing area, as fuel used during the hauling of creels is almost insignificant.

Similar results, in terms of comparative returns by trawls and creels, were found in Sweden [3], [28] where advantages in replacing bottom trawling with creels were reported due to less seafloor impact, less fish by-catch and undersized *Nephrops* discarded and a decrease in fuel consumption. In the Adriatic Sea, creels were found to be more ecologically sustainable due to lower discard levels, but were also shown to have lower economic viability due to the high scavenger activity in the area, that reduces the lifespan of the bait [4].

The maintenance of a trawl fishery for *Nephrops*, of apparently little economic interest, can be understood with a global analysis of this multi-target fishery. Nowadays, the rose shrimp has become the main target species, due to remarkably high first sale prices (up to 200€ per Kg at certain times of the year). This species provides most of the economic return of the crustacean trawling fleet (around 70% from 1996 to 2007, DGPA database).

Given the economic interest of fishing *Nephrops* with creels, one might expect the increase of this activity. At present, the biggest limitation for the expansion of the creel fishery in Portugal is gear conflict. Trawlers operate outside the 6 miles limit and *Nephrops* is uncommon within the 6 miles limit. Creel fishing is therefore restricted to sheltered areas where trawlers cannot operate, due to irregular and rocky bottoms. There are not many of these areas and territorialism or lack of information among fishermen might also hold back the expansion of creel use. This situation limits the proportion of fishing effort allocated by the artisanal fleet to *Nephrops* creel fishing.

Considering the current condition of the Portuguese *Nephrops* populations in the South and Southwest coasts (under a recovery plan) and the negative impacts that trawling has on the environment, creels may provide a more sustainable and profitable alternative to trawls, contributing to the recovery of the resource. This could be achieved without detrimentally affecting the rose shrimp fishery that occurs on the slope at shallower depths than *Nephrops* [32] and almost exclusively on the South coast. A transition regime could include, for example, the exclusion of trawlers from the SW coast, leaving the deep water crustaceans (including *Nephrops*) to be exploited by creels, while maintaining the present regime on the South coast (Figure 2). This would imply a reduction in the number of trawlers overall, to avoid relocation of trawl fishing effort to the South coast. Examples of the coexistence of both sectors can be found in Scotland, with different levels of conflict [25] and informal assignment of fishing grounds to both gears.

This is a preliminary study and consequently there are still large gaps in the present knowledge of how *Nephrops* creel fisheries exploit the stock and how this varies in different fishing areas. In this study it was not possible to compare the exploitation pattern of both gears directly, as trawling and creeling take place in different areas and exploit different *Nephrops* populations. Future studies should also include demand and supply analyses, to evaluate the needs of the market and changes that might occur from switching from trawls to creels such as decrease in total catch and increase in the fraction of high value lobsters. Sensitivity analysis could usefully be supplemented by formal risk analysis in order to generate probability distributions of the NPV outcomes.
